# Bacteroides *fragilis *aortic arch pseudoaneurysm: case report with review

**DOI:** 10.1186/1749-8090-3-29

**Published:** 2008-05-20

**Authors:** Hsin-Ling Lee, Kung-Hung Liu, Yu-Jen Yang, Chung-Dann Kan

**Affiliations:** 1 Institute of Clinical Medicine and Cardiovascular Research Center, Medical College, National Cheng Kung University, 1 University Road, Tainan City, 70101 Taiwan, Republic of China; 2 Department of Occupational and Environmental Medicine, National Cheng Kung University Hospital, 138 Sheng-Li Road, Tainan, Taiwan 704, Republic of China; 3 Department of Medicine, National Cheng Kung University Hospital, 138 Sheng-Li Road, Tainan, Taiwan 704, Republic of China; 4 Department of Surgery, National Cheng Kung University Hospital, 138 Sheng-Li Road, Tainan, Taiwan 704, Republic of China

## Abstract

We present a case of 58-year-old woman with underlying diabetes mellitus, hepatitis C virus-related liver cirrhosis, and total hysterectomy for uterine myoma 11 moths ago, who was diagnosed ruptured aortic arch mycotic pseudoaneurysm after a certain period of survey for her unknown fever cause. After emergent surgery with prosthetic graft interposition, all her blood cultures and tissue cultures revealed pathogen with *Bacteroides fragilis*. Although mycotic aneurysms have been well described in literatures, an aneurysm infected solely with *Bacteroides fragilis *is unusual, with only eight similar cases in the literature. Here we reported the only female case with her specific clinical and management course and summarized all reported cases of mycotic aneurysm caused by *Bacteroides fragilis *to clarify their conditions and treatments, alert the difficulty in diagnosis, and importance of highly suspicious.

## Introduction

Aortic mycotic aneurysm of the thoracic aorta is a rare but fulminant infectious disease and may potentially progress to rupture and death unless early diagnosis and appropriate treatment is instituted [1,2]. The early case reports emphasized endocarditis as the most common source, while hematogenous seeding, direct spreading from a contiguous focus with trauma, lymphatic spreading, and unknown etiology were proposed [1,3,4]. *Staphylococcus aureus*, nontyphi *Salmonella*, and *Pseudomonas species *have been implicated for most causative organisms [1,4]. After the era of antibiotics, the epidemiology of this disease is changing. *Bacteroides fragilis *was reported as a rare causative pathogen. We describe a case of *B. fragilis *aortic arch mycotic pseudoaneurysm in a female patient who presented with fever of unknown origin (FUO).

## Case report

A 58-year-old woman with diabetes mellitus, hepatitis C virus-related liver cirrhosis, and total hysterectomy for uterine myoma was admitted to another hospital because of a one-month history of recurrent fevers. Blood cultures were all negative, and a CT scan of the abdomen and pelvis was unremarkable. After a week of intravenous antibiotic treatment, she still presented with mild fever. Owing to that persisted intermittent low-grade fever, she was transferred to our institution and admitted for her fever cause surveying.

**Figure 1:**
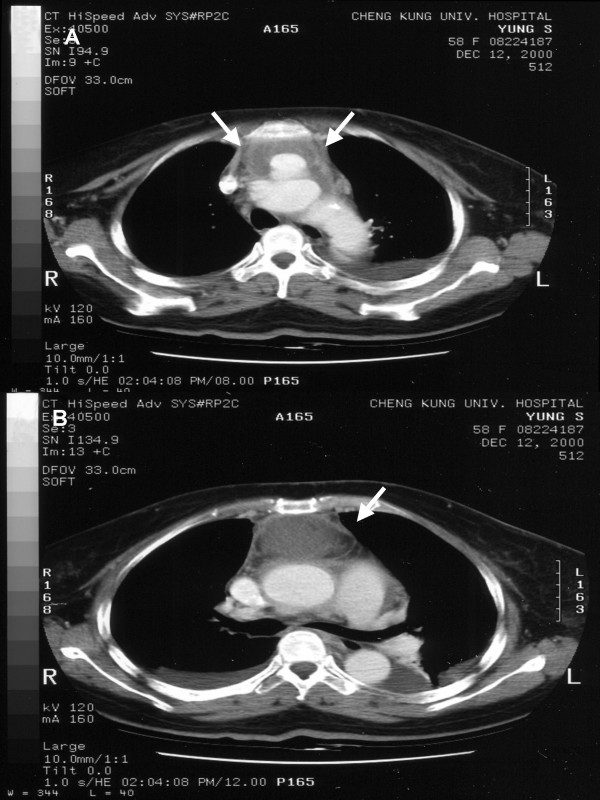
Axial CT scans. (A, B) Images of upper mediastinum show pseudoaneurysm with periaortic infiltration (white arrow).

At admission, she complained of aching sensation on her precordial area while coughing in recent one week. Her initial vital signs revealed a high fever up to 39.5°C, blood pressure of 140/88 mmHg, heart rate of 115 beats/min, and tachypnea of 28/min. The physical examinations were remarkable only for pale conjunctivae and crackles at the right lung base. Laboratory studies showed leukocytosis of WBC count 11,800/μL (74% neutrophils, 13% band forms, 7% lymphocytes, 5% monocytes, and 1% basophils); hemoglobin level 10.8 g/dL; and platelet count 102,000/μL. The C-reactive protein concentration was 182 mg/L. Electrolyte levels and renal function test results were within normal limits. The chest radiography revealed a mildly widened mediastinum with bilateral blurred costovertebral angles. Chest and abdominal computed tomography disclosed a mycotic pseudoaneurysm originating from the aortic arch with upper mediastinitis (Fig. 1). The transthoracic echocardiogram revealed no evidence of infective endocarditis. Empirical treatment with cefotaxime and teicoplanin was administrated parenterally, and she was transferred to the intensive care unit for further care. The immediate aorta-coronary angiogram survey also confirmed an aortic arch aneurysm with normal coronary vessels. Suddenly, she was noted with paradoxical pulse on the blood pressure monitor. Owing to exacerbation of dyspnea, accumulation of massive left pleural effusion with tracheal deviation to the right side, and an enlarged heart shadow on the follow-up chest roentgenogram, the patient received an emergent operation under the suspicion of ruptured mycotic pseudoaneurysm. The operation was performed by deep hypothermia and circulatory arrest with superior vena cava retrograded brain protection. A ruptured mycotic pseudoaneurysm in the arch region (apparent orifice between the innominate artery and the left common carotid artery), diffuse mediastinal abscess, and pericardial effusion were found at operation. Ascending aorta-to-aortic arch prosthetic graft interposition (Meadox™ Hemashield^® ^collagen graft) with innominate artery reimplantation were performed smoothly. Later, her blood cultures and resected tissue cultures all yielded *B. fragilis *were noticed, so the antibiotic regimen was adjusted according to the microbiological results. However, progressive jaundice with hepatic function impairment developed after surgery. Hemodynamic instability due to paroxysmal atrial fibrillation and rapid ventricular response and deterioration of consciousness occurred later. Intermittent low-grade fever developed again. Repeated blood cultures on postoperative day 10 revealed *Candida albicans*, and then amphotericin B was prescribed. Even though under intensive management and antimicrobial therapy, her hepatic and renal function continued to deteriorate and she died of multiple organ failure on postoperative day 14.

## Discussion

Although the first reported mycotic aneusym was introduced in 1885 by Sir Willam Osler for fungal vegetations in the aortic arch complicated by endocarditis, mycotic aneurysm remains one of the most life-threatening conditions in the field of vascular surgery. The prevalence of the mycotic type among all forms of aortic aneurysm is estimated about 1–2.7% [1,5]. The most common infection sites are the femoral artery and abdominal aorta, followed by the thoracoabdominal and thoracic aorta [1,6]. Essentially, three mechanisms of mycotic aortic aneurysm have been implicated, namely, septic embolization that usually is secondary to bacterial endocarditis; direct or lymphatic spread from an adjacent infected focus; and hematogenous seeding of the arterial wall during bacteremia from a distant focus [1,3,4].

*B. fragilis *is one of the normal floras in human terminal ileum, colon, and vagina, but it is also a major anaerobic pathogen to cause serious infections and attribute to high mortality if the normal intestinal mucosal is breached, especially in man. Our patient presented with episodes of fever that were suppressed with antibiotic therapy but recurred quickly once treatment was withdrawn. She had undergone vaginal total hysterectomy for uterine myoma complicated by pelvic abscess formation 11 months ago. Although abscess had been drained, we believed this lesion might be the source of the *Bacteroides *bacteremia. In spite of mycotic aneurysms have been well described in literatures, an aneurysm infected solely with *B. fragilis *is unusual. In the literatures, there are total only nine case reports of a similar process with variant locations, clinical presentations and possible etiologies [7-13] (Table 1). Summary from their demography, mostly, this disease happens on men, except for our patient. Most of them were pseudoaneurysm except one, when diagnosed. An thoracic mycotic aneurysm usually is suspected only when mediastinum widening is found on a chest film or incidentally during a survey CT scan [1,14]. Even under aggressive anaerobic cultures there still may miss a significant number of bacteremias like this strain and owing to there are no significant clinical findings that are pathognomonic of this disease and the laboratory studies usually show nonspecific results, diagnosis is often delayed.

**Table 1:** Reported cases of mycotic aneurysm infected by *Bacteroids fragilis*

**Author, year**	**Age, Sex**	**Lo**	**Clinical presentation**	F*	**Possible etiology**	**Find-ing**	**Surgery**	**Results**
Present case	58, F	Arch	FUO	Y	Pelvic abscess	PA	Graft	Expired, 14 days
Beland, 2005	65, M	Da	Hemoptysis	N	Diverticulitis, leprosy	PA	EVAR	Survived
Tsuji 2003	74, M	Iliac	Chronic back pain	N	Osteomyolitis	PA	Survived	
Matsuyama 2003	69, M	Da	Sudden back pain	N	Cholecystitis	PA	Survived	
Doita 2001	60, M	Aa	Low back pain	N	Pyogenic osetomyolitis	PA	Survived	
O'Donnell 1999	71, M	Aa	FUO	Y	Unknown	SA	Yes, ND	Expired, on table
Jewkes 1989	58, M	Aa	Abdominal pain	Y	Appendiceal abscess	SA	Extra anatomic bypass	Survived
Sheehan 1983	53, M	Aa	Pulsatile mass	Y	Translumbar aortography	PA	Laparotomy	Expired

Aa: abdominal aorta; Da: descending aorta; FUO: fever of unknown origin; ND: not described; PA: pseudoaneurysm; SA: saccular aneurysm

The conventional strategy for the treatment of mycotic aneurysm is prompt surgical intervention followed by long term antibiotic therapy, which is essential to control systemic sepsis and to achieve cardiovascular stability. Antibiotics alone are not sufficient, and complete excision of the affected aorta is the key to curative treatment [1,10,14]. However, the surgical procedures are associated with substantial mortality rates associated with the risk of recurrent infection and the survival was influenced not by the type of reconstruction but by the status of aneurismal rupture [11]. The use of homograft, antibiotic-coated grafts to reduce the source of infection, or of a coated endoprosthesis to release antibiotics into the blood stream, have been proposed for the successful management [15]. However, it depends on the availability of hospital. Several authors advocated for endovascular stent-graft treatment with no mortality in small case reports [15]. The main advantages of this minimally invasive approach are the reduction of surgical trauma as well as minimal hemodynamic alterations. It may ultimately become the standard of care if results prove equivalent to open intervention. Even though, the difficult application in ascending aorta to arch region, the possibility of stent graft infection, and the unaffordable product prices are major considerations for their usage. In addition, fever presentation (3/4,75%), indicated active process persisted, in such patients seems a terrible signature for most of patients would have poor prognosis even under aggressive treatment.

In conclusion, it should be noted that *Bacterioides fragilis *is a rare causative pathogen and the primary source of this bacterium is often undetermined. A higher clinical awareness of this disease, leading to early computed tomography evaluation and prompt surgical intervention under appropriate and intensive antibiotic therapy, appears to offer the best chance of survival in patients with this difficult condition.
